# Functional identification of purine permeases reveals their roles in caffeine transport in tea plants (*Camellia sinensis*)

**DOI:** 10.3389/fpls.2022.1033316

**Published:** 2022-12-15

**Authors:** Yazhen Zhang, Kang Wei, Lingling Guo, Yuping Lei, Hao Cheng, Changsong Chen, Liyuan Wang

**Affiliations:** ^1^ Key Laboratory of Tea Biology and Resources Utilization, Ministry of Agriculture, National Center for Tea Improvement, Tea Research Institute Chinese Academy of Agricultural Sciences (TRICAAS), Hangzhou, China; ^2^ Tea Research Institute, Fujian Academy of Agricultural Sciences, Fuzhou, China

**Keywords:** caffeine, transport, purine permease, functional identification, tea plant

## Abstract

Caffeine is a characteristic secondary metabolite in tea plants. It confers tea beverage with unique flavor and excitation effect on human body. The pathway of caffeine biosynthesis has been generally established, but the mechanism of caffeine transport remains unclear. Here, eight members of purine permeases (PUPs) were identified in tea plants. They had diverse expression patterns in different tissues, suggesting their broad roles in caffeine metabolism. In this study, F1 strains of "Longjing43" ♂ × "Baihaozao" ♀ and different tea cultivars were used as materials to explore the correlation between caffeine content and gene expression. The heterologous expression systems of yeast and Arabidopsis were applied to explore the function of CsPUPs. Correlation analysis showed that the expressions of CsPUP1, CsPUP3.1, and CsPUP10.1 were significantly negatively correlated with caffeine content in tea leaves of eight strains and six cultivars. Furthermore, subcellular localization revealed that the three CsPUPs were not only located in plasma membrane but also widely distributed as circular organelles in cells. Functional complementation assays in yeast showed that the three CsPUPs could partly or completely rescue the defective function of *fcy2* mutant in caffeine transport. Among them, transgenic yeast of CsPUP10.1 exhibited the strongest transport capacity for caffeine. Consistent phenotypes and functions were further identified in the CsPUP10.1-over-expression Arabidopsis lines. Taken together, it suggested that CsPUPs were involved in caffeine transport in tea plants. Potential roles of CsPUPs in the intracellular transport of caffeine among different subcellular organelles were proposed. This study provides a theoretical basis for further research on the PUP genes and new insights for caffeine metabolism in tea plants.

## 1 Introduction

Caffeine, namely, 1,3,7-trimethylxanthine, is an important functional metabolite in plants and has effects of excitement and diuresis on human body (Shilo et al., 2002; Celik et al., 2010; Gramza-Michalowska, 2014). It is a common component in tea (*Camellia sinensis*), coffee (*Coffea arabica* and *Coffea canephora*), and cocoa (*Theobroma cacao*), which were widely consumed as the three major soft drink in the world. However, the distribution of caffeine in tea plants is greatly distinct from coffee and cocoa. Caffeine in tea plants mainly occurred and accumulated in leaves, rather than in seeds and fruits. Moreover, its content in young tea leaves is 2%–3% of dry weight and even higher than that in coffee (Suzuki et al., 1992; Ashihara and Suzuki, 2004). The subcellular distribution of its biosynthesis is suggested to be localized in the chloroplasts, where *de novo* and salvage synthesis pathway occur and most key biosynthetic enzymes exist (Ashihara et al., 2013). Whereas, caffeine in coffee appears to be synthesized in the cytoplasm (Ogawa et al., 2001; Kumar et al., 2007). The vacuole was supposed to be the caffeine storage site, which was similar to other water-soluble secondary metabolites in plants (Wink, 2010). For now, the pathway of caffeine biosynthesis has been generally established (Ashihara et al., 2013; Jin et al., 2016). However, studies on its transport process have not been reported yet.

The mechanisms of alkaloids transport in some plants have been revealed (Shitan et al., 2014). Alkaloid transporters play an essential role in maintaining the dynamic equilibrium of alkaloids in different types of organelles, cells, and tissues. Purine permeases (PUPs) were recently reported to be involved in the transportation of purine, pyridine, tropane, and benzylisoquinoline alkaloids, as well as their derivatives and analogs (Jelesko et al., 2012; Dastmalchi et al., 2019).

PUPs are found specifically in vascular plants and assigned to an ancient drug and metabolite transporter super family (Jelesko, 2012). For the past decade, PUPs were primarily found to uptake purine nucleobase and their derivatives. The function was first demonstrated in Arabidopsis (Gillissen et al., 2000). In total, 21 PUP members were found in Arabidopsis. They were predicted to contain typically 9–10 transmembrane spanning domains (Qi and Xiong, 2013). AtPUP1 and AtPUP2 showed high sequence identity and similar substrate specificity. They were proved to be energy-dependent and H^+^-coupled transporters of adenine and cytokinin. Their different tissue-specific expression patterns indicated distinctive transport mechanisms in Arabidopsis. An expanding role of AtPUP1 and AtPUP2 in caffeine transport in yeast was also proved. However, none of transport activity was detected in AtPUP3 yeast transformants (Gillissen et al., 2000; Bürkle et al., 2003). Further study on AtPUP1 revealed that it also showed high transport activity for pyridoxine (vitamin B6) (Szydlowski et al., 2013). AtPUP14 was localized on the plasma membrane and could import bioactive cytokinins to the cytosol (Zürcher et al., 2016). Twelve PUP members were found in rice (*Oryza sativa*). OsPUP7 was mainly expressed in the vascular bundle, pistil, and stamens. It was identified as caffeine transporter in yeast and played an important role in the cytokinin transportation in rice (Qi and Xiong, 2013). Further studies showed that OsPUP7 was localized on endoplasmic reticulum (ER) and positively regulated the grain size. The overexpression (OE) of OsPUP4, the closest homolog gene to OsPUP7, resulted in highly similar phenotypes. However, different from OsPUP7, OsPUP4 was localized on the plasma membrane. It was inferred that OsPUP4 and OsPUP7 function synergistically as cytokinin transporters in its long-distance movement and local allocation (Xiao et al., 2018). OsPUP1 was also localized on ER and could import cytokinins from the vascular tissues by cell-to-cell transport (Xiao et al., 2020). NtNUP1 (nicotine uptake permease), which belongs to PUPs family in tobacco, was demonstrated to transport various compounds in yeast system. Compared with pyridine alkaloids, tropane alkaloids, kinetin, and adenine, NtPUP1 showed preferential transport activities to nicotine. It revealed that NtPUP1 functioned as nicotine transporters in root tips from its synthesis sites (apoplastic space) into the cytoplasm (Hildreth et al., 2011; Kato et al., 2015). There are nine PUP members in opium poppy (*Papaver somniferum*), and six of them were identified as benzylisoquinoline alkaloid transporters. Each member showed a unique substrate preferential profile (Dastmalchi et al., 2019). Fifteen PUPs were found in *Coffea canephora*. CcPUP1 and CcPUP5 were demonstrated to facilitate the transport of adenine, but not caffeine in yeast uptake experiments (Kakegawa et al., 2019).

However, studies on PUP members or caffeine transporters in tea plants have not been reported. In this study, we aimed to (i) systematically investigate the genome-wide PUP genes in tea plants, including their bioinformatics analysis and expression patterns, (ii) explore the relationship between caffeine contents and CsPUP gene expression, and (iii) identify the function of CsPUPs in heterologous expression system, including yeast and Arabidopsis. This study could fill the research gap of caffeine transporters and provide a new insight into the regulation of secondary metabolism in tea plants.

## 2 Materials and methods

### 2.1 Plant materials

One-year-old cutting seedlings of “Zhongcha108” were used for the analysis of gene expression characteristics in different tissues, which was planted in the tea garden of the Tea Research Institute Chinese Academy of Agricultural Sciences (TRICAAS) in Hangzhou, China. Young leaves (YL) and mature leaves (ML) of different tea strains and cultivars were used for correlation analysis between caffeine content and gene expression. They grew for 4–5 years in the tea garden of TRICAAS in Shengzhou, China. Details of tea plant materials were as follows: F1 strains of “Longjing43” ♂× “Baihaozao” ♀, numbered as 502, 915, 1417, 1916, 1501, 1511, 1608, and 1912, were collected on 24 July 2019. Tea cultivars, including “Longjing43” (LJ), “Zhongcha108” (ZC), “Zhongming7” (ZM), “Zhongbai4” (ZB), “Zhonghuang1” (ZH), and “Enshi4” (ESN), were collected on 17 April 2020.

### 2.2 Bioinformatics analysis of CsPUPs

CsPUP members were screened by text search “purine permease” on the “Shuchazao” (*Camellis sinensis* var. sinensis) genome database (http://tpia.teaplant.org/index.html ). Then, the sequences of CsPUPs were submitted for BLASTX analysis with Arabidopsis in NCBI website (https://blast.ncbi.nlm.nih.gov/Blast.cgi ). Transmembrane regions of CsPUPs proteins were predicted by the online software TMHMM server v.2.0 (http://www.cbs.dtu.dk/services/TMHMM-2.0/ ). Amino acid sequence alignment of CsPUPs was performed by DNAMAN. The phylogenetic tree was constructed by MEGA 6.0 using the neighbor-joining method. The genomic information of CsPUPs was screened by local blast software (ncbi-blast-2.9.0+); then, the genomic locations of CsPUPs were determined by MapChart.

### 2.3 Determination of caffeine content

The measurement of caffeine content by HPLC was described by Zhang et al. (2020). Collected materials were dried and grinded into powders. Then, 0.2 g of samples were extracted with 5 ml of 70% (v/v) methanol at 70°C water bath for 10 min and centrifuged at 3,500×g for 10 min. The extraction was repeated to reach a final volume of 10ml supernatant. Then, they were filtered through 0.45-μm Millipore filters and subjected for high performance liquid chromatography (HPLC) analysis. Reverse-phase column (Phenomenex C12, 4.6 × 250 mm, 5 μm) was used for the component assay. The mobile phases were as follows: 1% formic acid (solvent A), 100% acetonitrile (solvent B), and ultrapure water (solvent D). The linear elution gradient was 0–42 min (4%–18.7% B) and 42–43 min (18.7%–4% B). The samples were measured at 280 nm and eluted at 1 ml min^−1^.

### 2.4 Gene expression analysis

First, total RNA extraction and cDNA synthesis were performed using the RNAprep Pure Plant Kit and FastQuant RT Kit (Tiangen, Beijing, China), respectively. Then, quantitative real-time (qRT)-PCR analysis was performed on a Roche LightCycler 480 II Real-Time PCR system using the PrimeScript RT reagent qPCR Kit (Takara, Dalian, China). All primers were designed by online software Primer-BLAST and displayed in Additional File 1. glyceraldehyde-3-phosphate dehydrogenase (CsGAPDH) was used as housekeeping gene. The relative gene expression level was calculated using the 2^−∆∆Ct^ method (Livak and Schmittgen, 2001).

### 2.5 Subcellular localization analysis of CsPUPs

The method of subcellular localization in tobacco epidermis was similar to that by Wang et al. (2022). The coding sequences (CDS) of CsPUP1, CsPUP3.1 and CsPUP10.1 without stop codon were amplified and cloned into the pBWA(V)HS-GFP vector, respectively. The specific primers were listed in Additional File 1. The plasmid of recombinant vector was transformed into *Agrobacterium* GV3101. The OsMCA1 was used as a plasma membrane (PM) marker for co-localization of target protein. 35S-GFP with empty vector (EV) was used as a positive control. Then, the transformant cells were injected into the lower epidermis of tobacco leaves and cultured for 48h under low light. Finally, the GFP signals were detected and recorded by confocal lase microscope (Nikon C2-ER).

### 2.6 Functional identification of CsPUPs in yeast

The homologous protein of PUP in yeast was encoded by FCY2 gene. It was first proved to transport purine-cytosine (Ferreira et al., 1997). The knock-out mutant *fcy2* was used as heterologous expression system for identifying transporters of nucleic acid bases and their derivatives in plants, such as cytokinin and caffeine. (Gillissen et al., 2000). It was also regarded as deficiency in caffeine transport and used for functional identification of PUPs in Arabidopsis and rice (Bürkle et al., 2003; Qi and Xiong, 2013). Therefore, to verify the function of CsPUPs, the gene CDS was cloned into the yeast-expression vector pYES2 by double enzymes digestion. Then, the plasmids with CsPUP1, CsPUP3.1, CsPUP10.1 and EV (control) were transformed into the yeast *fcy2* mutant (BY4741, *Mata, his3D1, leu2D0, met15D0, ura3D0*, YER056c::kanMX4). The normal WT yeast BY4741 was used as a positive control. The detailed method used the Quick and Easy Yeast Transformation Mix (Takara, Dalian, China). According to the information of pYES2 vector, the minimal medium lacking uracil was used as a basic medium. The medium containing 2% galactose and 1% raffinose as the carbon source was used as an inducing medium. The yeast cells were first activated in the basic medium plate for 2–3 days at 30°C. Then, single colony was pre-incubated overnight in the basic medium broth at 30°C with shaking at 200 rpm. The value of OD_600_ was measured and normalized to 0.5 by saline solution. Then, the suspension cells were diluted tenfold successively. Finally, 50 μl of a hundredfold dilution (10^−2^) was used for the yeast growth assay under 0.3% caffeine treatment (Qi and Xiong, 2013; Kakegawa et al., 2019).

For caffeine uptake assays in yeast, the method was slightly modified according to Morita et al. (2009) and Shoji et al. (2009). The OD_600_ value of pre-incubated yeast cells was normalized to 0.4 according to the pYES2 manual (www.invitrogen.com ). Then, yeast cells were centrifuged at 4,000 rpm for 10 min and washed by sterile water for three times. Then, cells were incubated in 40 ml of inducing medium broth for 24 h and continuously cultured under 0.3% caffeine treatment for another 24 h. Next, yeast cells were harvested, and their fresh weight was recorded. Two milliliters of 70% (v/v) methanol was added for disrupting the reaction and caffeine extraction. The cells were then disrupted by ultrasonication under 400–500 W for 5 min. The determination of the caffeine content was performed according to the method abovementioned (see **Section 2.3**).

### 2.7 Functional identification of CsPUP10.1 in Arabidopsis

The recombinant plasmid pBWA(V)HS-GFP-35S-CsPUP10.1 was transformed into Arabidopsis (WT, *Col-0*) by inflorescence infection method. Then, the homozygous transgenic plants were screened by hygromycin (25 μg/ml) on 1/2 Murashige and Skoog (MS) medium for three generations (Zhang et al., 2021). For the phenotypic comparison between transformants and control, Arabidopsis seeds were first sowed on normal 1/2 MS medium plate and grew for 7 days. Then, seedlings with consistent growth conditions were selected for caffeine treatment. They were transplanted to 1/2 MS medium containing 0.01%, 0.03%, and 0.05% caffeine and cultivated vertically for 14 days. For caffeine uptake assays in Arabidopsis, seeds were sowed on 1/2 MS medium containing 0.02% caffeine and grew for 21 days. Then, the aerial parts of plants were chosen for caffeine measurement. The method used was the same as the abovementioned (see **Section 2.3**).

### 2.8 Statistical analysis

The abovementioned samples or treatments were performed for three biological replicates. Significance analysis was conducted by a one-way analysis of variance using the Statistical Package for the Social Sciences Statistics 17.0. Different lowercase letters represent significant differences at a P-value < 0.05. A bivariate correlation analysis between gene expression and caffeine content was conducted using Pearson’s parametric correlation test. The figures were generated using the plotting software Origin 9.

## 3 Results

### 3.1 Identification and molecular characterization of CsPUPs in tea plants

**Figure 1:**
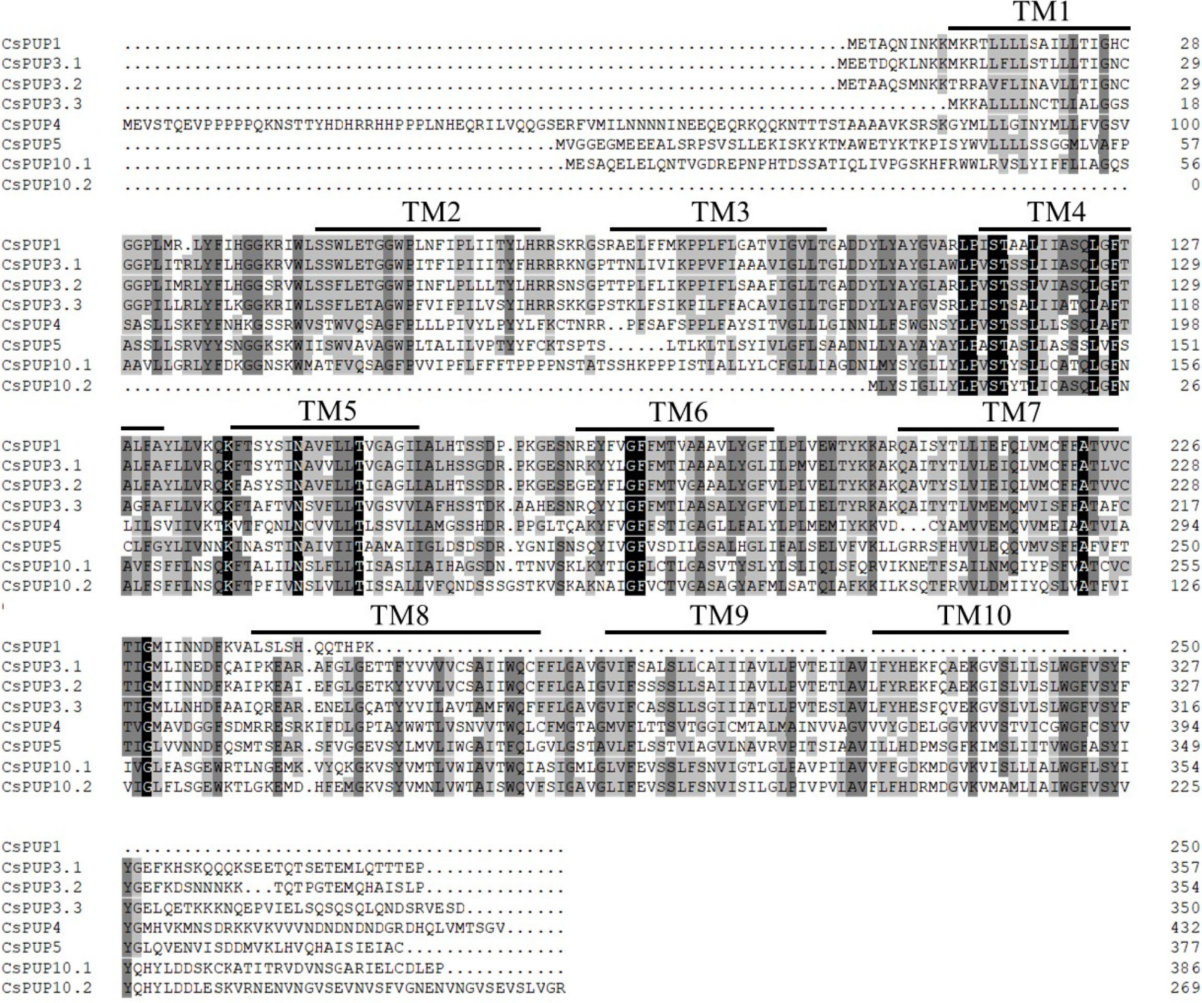
Amino acids sequences alignments of CsPUPs.

On the basis of the tea genome database of “Shuchazao”, eight CsPUP members were identified. The length of CDS was 723–1352 bp. Then, BLASTX analysis with Arabidopsis was conducted. The eight CsPUPs showed high sequence similarity (54%–73%) with five AtPUPs and were named as CsPUP1, CsPUP3.1, CsPUP3.2, CsPUP3.3, CsPUP4, CsPUP5, CsPUP10.1, and CsPUP10.2, respectively (**Table 1**). All of the CsPUP members have consensus conserved domains (purine nucleobase transmembrane, CL0184). The similarity of their amino acid sequences was 43.09%. Transmembrane structure analysis by TMHMM software showed that most of the CsPUPs contained 10 transmembrane domains (TMDs), except for CsPUP1 and CsPUP10.2. Further multiple alignment analysis showed that the sequence of TMDs was more conserved, especially for the TM4-TM7 (**Figure 1**).

**Table 1:** Identification of PUPs in tea plants.

Name	Gene ID	CDS length	Homology	Similarity
CsPUP1	TEA003596	837	AtPUP1	59.46%
CsPUP3.1	TEA029223	1074	AtPUP3	63.07%
CsPUP3.2	TEA003576	1065	AtPUP3	59.45%
CsPUP3.3	TEA013066	1053	AtPUP3	56.67%
CsPUP4	TEA015426	1299	AtPUP4	64.49%
CsPUP5	TEA031626	1134	AtPUP5	73.16%
CsPUP10.1	TEA023430	1161	AtPUP10	53.93%
CsPUP10.2	TEA002721	813	AtPUP10	70.13%

http://tpia.teaplant.org/download.html

**Figure 2:**
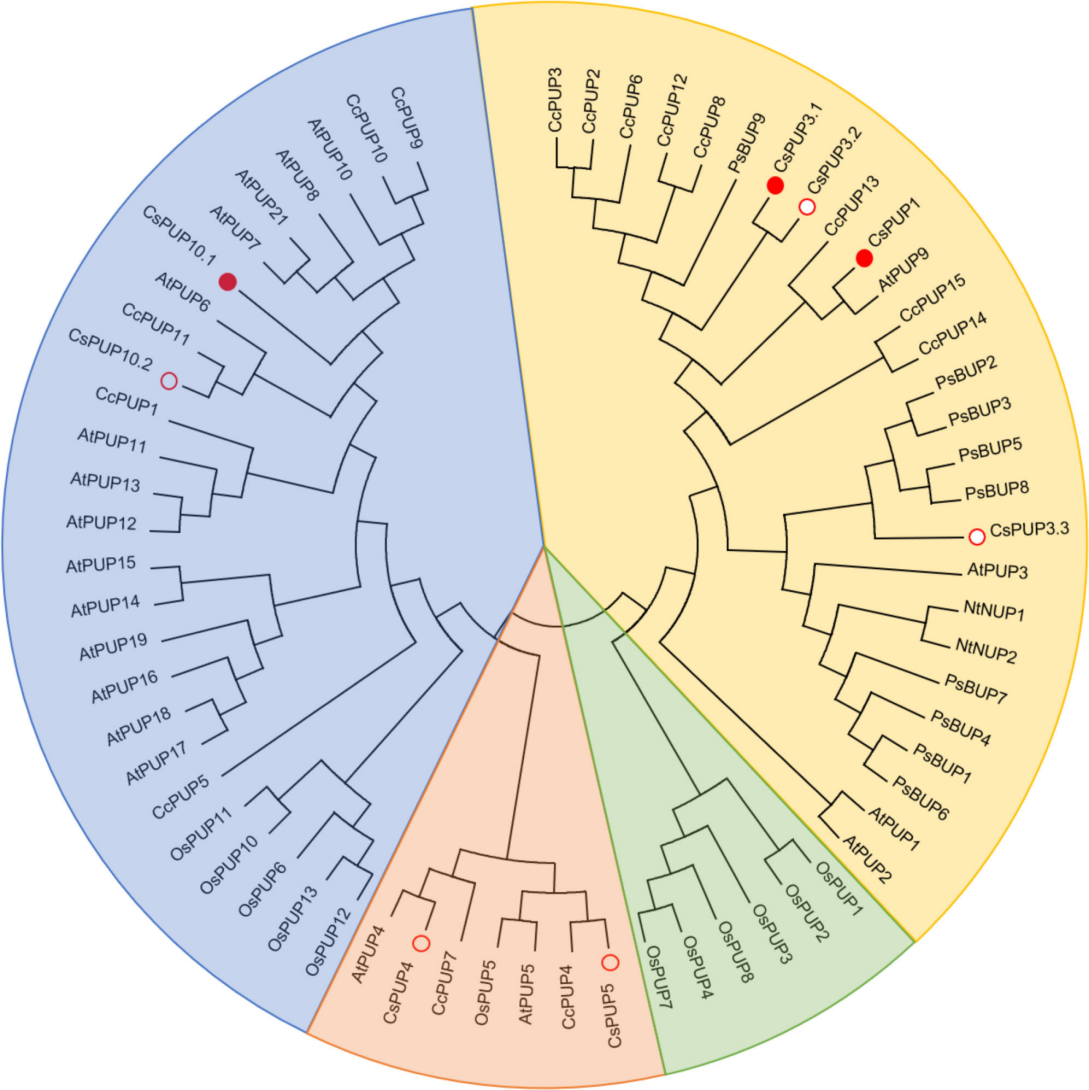
Phylogenetic tree analysis of PUP family members.

To elucidate the phylogenetic relationships among PUPs in *Camellia sinensis*, *Arabidopsis thaliana*, *Oryza sativa*, *Nicotiana tabacum*, *Coffea canephora*, and *Papaver somniferum*, a phylogenetic tree was constructed by aligning eight CsPUPs, 20 AtPUPs, 13 OsPUPs, two NtPUPs, 15 CcPUPs, and nine PsBUPs using MEGA6.0 (**Figure 2**). Phylogenetic analysis revealed that these 67 PUPs in the six species could be clustered into four subgroups. CsPUPs were distinctly distributed into three clades: CsPUP1, CsPUP3.1, CsPUP3.2, and CsPUP3.3 as clade I; CsPUP4 and CsPUP5 as clade II; and CsPUP10.1 and CsPUP10.2 as clade III.

**Figure 3:**
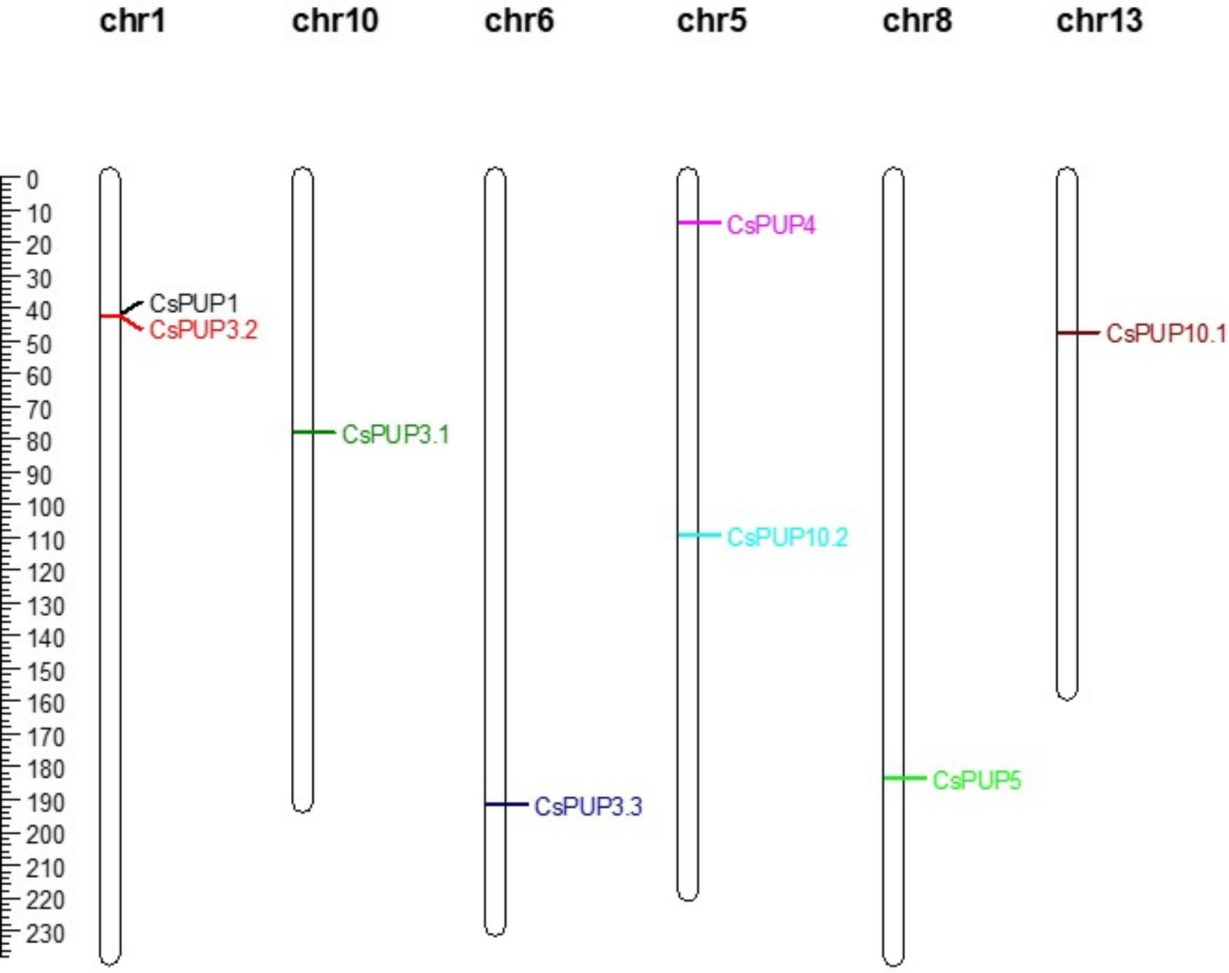
Distribution of the CsPUPs genes on chromosomes.

Through the physical position identification, eight CsPUPs were unevenly distributed on six chromosomes (**Figure 3**). CsPUP1 and CsPUP3.2 were located on the chr1. CsPUP4 and CsPUP10.2 were located on the chr5. CsPUP3.1, CsPUP3.3, CsPUP5, and CsPUP10.1 were located on the chr10, chr6, chr8, and chr13, respectively.

### 3.2 Expression patterns of CsPUPs in different tissues

**Figure 4:**
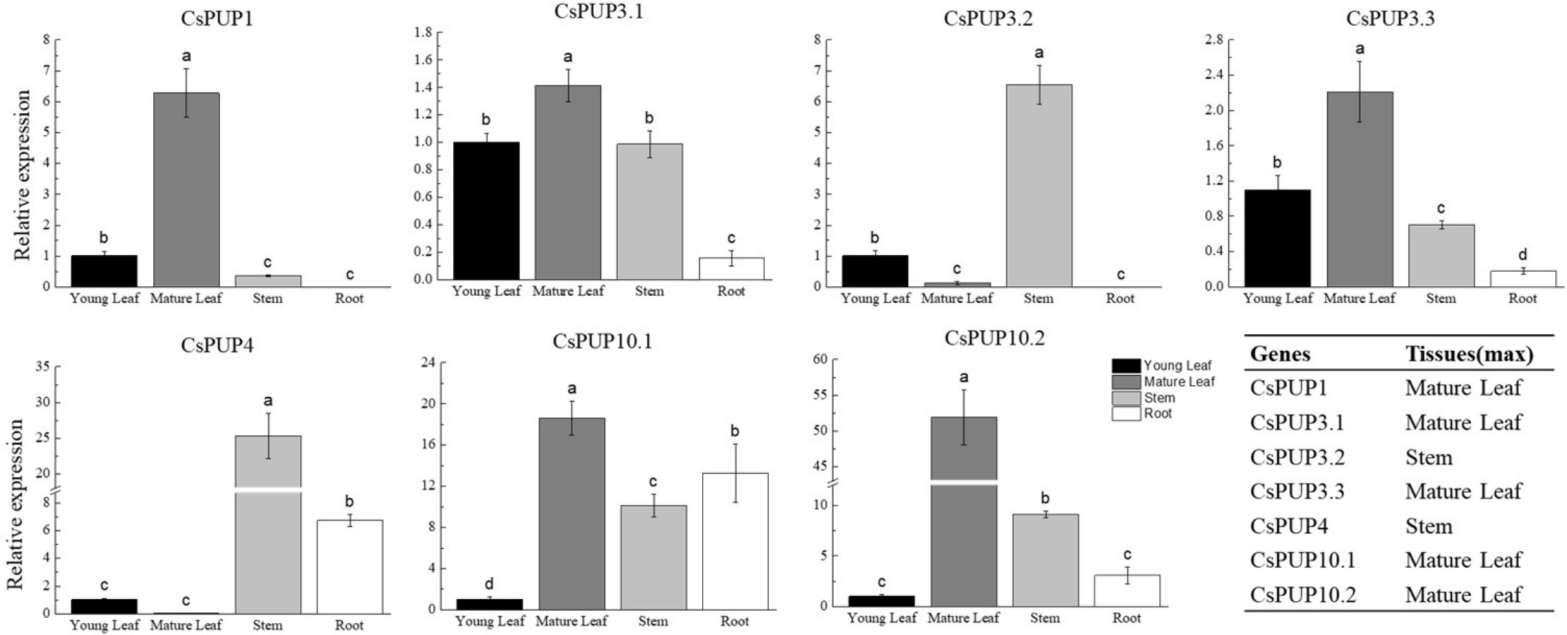
Expression patterns of CsPUPs in different tissues.

qRT-PCR was applied for the expression analysis of CsPUPs in different tissues, including young leaf (one bud with one leaf), mature leaf (the fifth leaf), stem, and root (**Figure 4**). CsPUP1 was mainly expressed in the mature leaf. CsPUP3.1, CsPUP3.3, CsPUP10.1, and CsPUP10.2 were expressed in the all tissues tested. CsPUP3.1 and CsPUP3.3 showed similar pattern and were primarily expressed in the aerial parts of tea plants. CsPUP10.1 and CsPUP10.2 were highly expressed in the mature leaf. Interestingly, CsPUP3.2 and CsPUP4 were mainly expressed in the stem, lower or no detectable expression in leaves. CsPUP5 was failed to be amplified in this study. The results revealed that these CsPUPs have tissue-specific or preferential expression patterns. In general, CsPUP1 showed the highest expression level in the aerial parts, followed by CsPUP3.1, CsPUP3.3, and CsPUP10.1 (Additional File 2). In addition, CsPUP3.1 and CsPUP3.3 were clustered into clade I in phylogenetic tree (**Figure 2**) and showed similar expression model in different tissues (**Figure 4**). Considering that caffeine is mainly distributed in leaves, CsPUP1, CsPUP3.1, and CsPUP10.1 were selected for further investigation in this study.

### 3.3 Caffeine contents in different tea strains and cultivars

**Figure 5:**
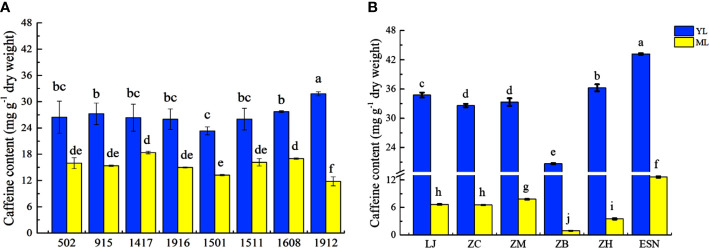
Caffeine content in different tea strains **(A)** and cultivars **(B)**.

Previous study proved that caffeine accumulation in tea leaves with different maturities varied greatly (Mohanpuria et al., 2009a). Gene expression levels of CsPUPs also showed obvious differences in YL and ML. Therefore, YL and ML were used for determining the correlation between gene expression and caffeine content. To investigate the caffeine accumulation in closely related and unrelated materials, eight strains of F1 population of plant hybrids from tea cultivars of “Longjing43” ♂× “Baihaozao” ♀, and six different cultivars were used in this study. The levels of caffeine ranged from 11.81 to 31.80 mg/g in the strains and from 0.92 to 43.18 mg/g in the cultivars, respectively (**Figure 5**). Similar caffeine accumulation patterns were found in different tea strains and cultivars. Caffeine content in YL was significantly higher than that in ML, which was consistent with the previous reports (Mohanpuria et al., 2009a). In addition, compared with the eight strains, greater variations of caffeine accumulation between YL and ML were observed in the six cultivars, especially for ZB (22.56 folds).

### 3.4 CsPUP gene expression and correlation with caffeine contents

**Figure 6:**
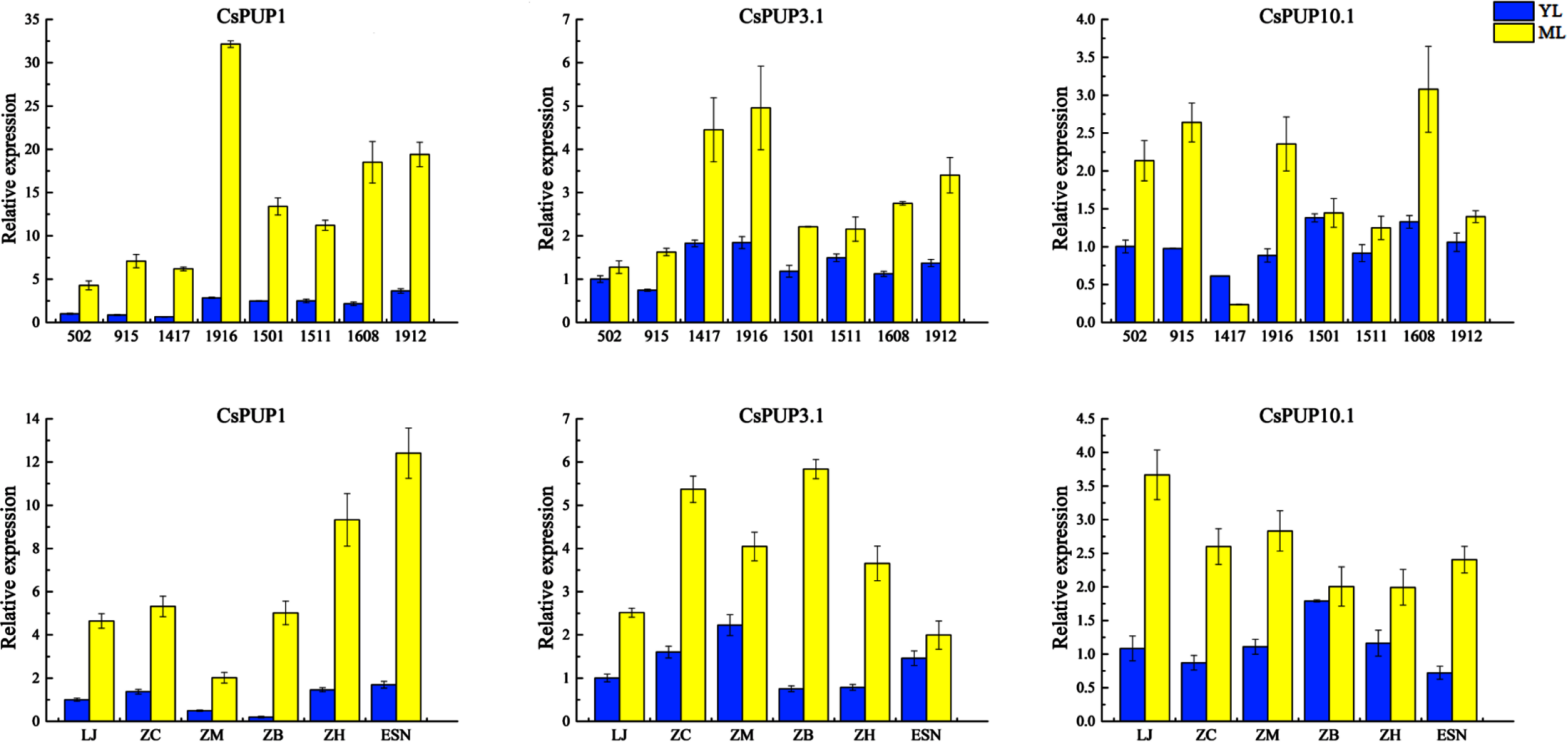
Expression levels of CsPUP1, CsPUP3.1 and CsPUP10.1 in different tea strains and cultivars.

The expression levels of CsPUP1, CsPUP3.1, and CsPUP10.1 in the abovementioned materials were analyzed by qRT-PCR (**Figure 6**). Consistent expression patterns were identified among the three genes. Overall, CsPUPs were expressed higher in ML than that in YL, regardless of the tea strains or cultivars. Among of them, CsPUP1 showed greater variations, 4- 25-fold changes were found in ML and YL.

To further explore the relationship between CsPUPs and caffeine content, correlation analysis was performed. The results revealed that the three gene transcription levels were significantly negatively correlated with caffeine concentration (**Table 2**). CsPUP10.1 showed the highest correlation coefficient with caffeine in different cultivars (−0.834^**^). Whereas in the eight strains, CsPUP1 was found to have the closest correlation with caffeine (−0.710^**^). These results suggested that these CsPUPs might play important roles in caffeine metabolism.

**Table 2:** Correlation analysis of caffeine content and CsPUPs expression.

Correlation	Eight strains	Six cultivars
CsPUP1	−0.710**	−0.615*
CsPUP3.1	−0.593*	−0.776**
CsPUP10.1	−0.524*	−0.834**

“*” and “**” represent the significance level at p < 0.05 and p < 0.01, respectively.

### 3.5 Subcellular localization of CsPUPs

**Figure 7:**
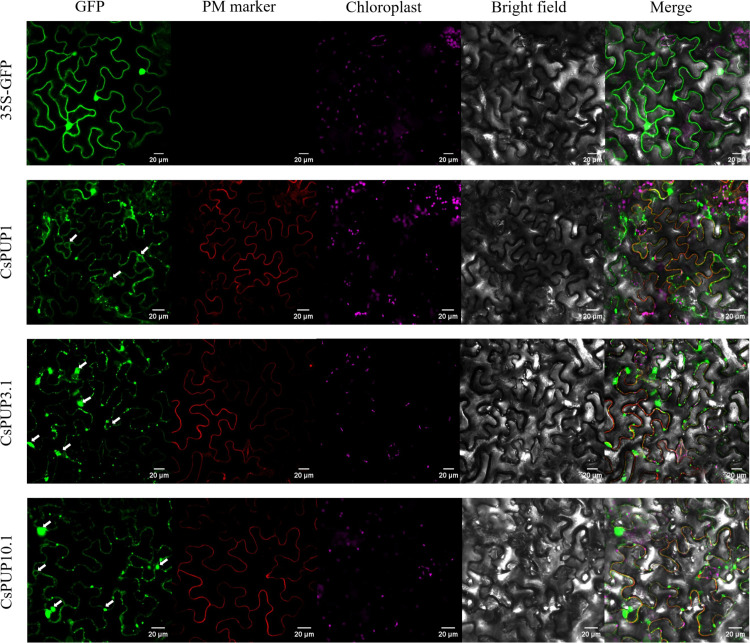
Subcellular localization of CsPUP1, CsPUP3.1 and CsPUP10.1.

The function of these transporters strongly depends on their distributions in cells. To confirm subcellular localization of CsPUP1, CsPUP3.1, and CsPUP10.1, full length of each gene was translationally fused with GFP. As shown in **Figure 7**, CsPUP1, CsPUP3.1, and CsPUP10.1 showed similar distribution patterns in the epidermic cells. The green fluorescence signals could coincide with the red fluorescence probe of PM marker protein, indicating that these CsPUPs are PM-associated protein. In addition, strong signals emerged as solid or hollow green dots were also detected. The dots irregularly distributed throughout the cell (white arrows in **Figure 7**). They are presumed to be secreted vesicles, peroxisomes, or lysosomes, which may contribute to performing the transport function of CsPUPs.

### 3.6 Functional identification of CsPUPs in yeast

**Figure 8:**
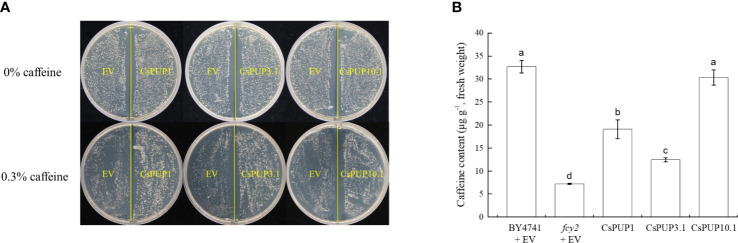
Functional identification of CsPUP1, CsPUP3.1 and CsPUP10.1 in yeast. **(A)** Phenotypic differences of yeast growth; **(B)** Caffeine accumulation in different yeast cells under 0.3% caffeine treatment.

The yeast mutant *fcy2* was defective in caffeine transport (Gillissen et al., 2000) and usually used for the functional identification of alkaloid transporters. For species without any caffeine produced, such as Arabidopsis and rice, the OE of PUPs in the yeast mutant *fcy2* was reported to facilitate the transport of caffeine. It provided initial and indirectly experimental evidence for gene function in cytokinins or other derivatives transport (Bürkle et al., 2003; Qi and Xiong, 2013; Kakegawa et al., 2019). For tea plants with abundant caffeine accumulation, the *fcy2* mutant was ideal system for the functional identification of CsPUPs. To determine whether CsPUPs have similar function, CsPUP1, CsPUP3.1, and CsPUP10.1 were individually cloned into the pYES2 vector. Then, the recombinant plasmids were transformed into the yeast mutant *fcy2*. On the medium without caffeine treatment, there was no significant difference in yeast growth between EV and transgenic yeast. Whereas on the medium with 0.3% caffeine treatment, the *fcy2* yeast transformed with CsPUPs grew better than EV (**Figure 8A**). It suggested that CsPUP1, CsPUP3.1, and CsPUP10.1 mediated caffeine uptake into yeast cells.

To further assess their caffeine transport capacities, yeasts were incubated on inducing medium for 24 h and then treated with 0.3% caffeine for another 24 h. The contents of caffeine in these yeast cells were then determined by HPLC. As shown in **Figure 8B**, the caffeine accumulation in different yeast cells was ranked as follows: (BY4741 + EV) > CsPUP10.1 > CsPUP1 > CsPUP3.1 > (*fcy2* + EV). The uptake of caffeine was significantly higher in the transgenic cells than that in the negative control (*fcy2* + EV). Compared with *fcy2*, the caffeine transport capacity of CsPUP1, CsPUP3.1, and CsPUP10.1 transgenic cells increased for 2.7, 1.7, and 4.2 folds, respectively. In addition, caffeine content in normal wild-type (WT) yeast BY4741 was 4.6 folds higher than that in *fcy2* mutant, which confirmed the defective function of *fcy2* mutant. No significant difference was observed in caffeine uptake between BY4741 and CsPUP10.1. These results indicated that CsPUP1, CsPUP3.1, and CsPUP10.1 could partly or completely rescue the caffeine transport deficiency of *fcy2* mutant and function as caffeine influx transporters. Moreover, yeast transformed with CsPUP10.1 exhibited the strongest capacity of caffeine transport and was used for further investigation in this study.

### 3.7 Functional identification of CsPUP10.1 in Arabidopsis

**Figure 9:**
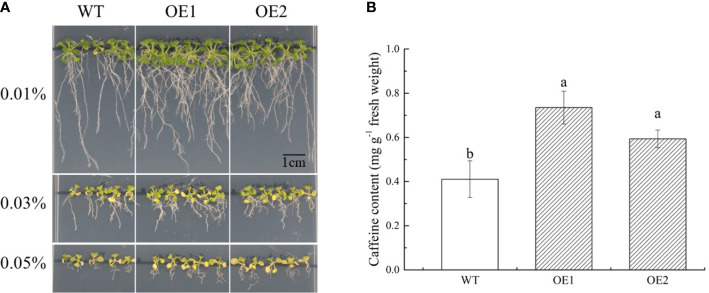
Functional identification of CsPUP10.1 in Arabidopsis. **(A)** Phenotypic differences of transgenic over-expression (OE) lines and wild type (WT) under caffeine treatments. **(B)** Caffeine accumulation in Arabidopsis under 0.01% caffeine treatment.

To further clarify the function of CsPUP10.1 in caffeine transport and accumulation in plant, the gene was transformed into Arabidopsis for generating OE lines. Then, two transgenic lines (OE1 and OE2) and WT (control) were selected for exogenous caffeine treatment. On 1/2 MS medium without caffeine, no significant phenotypic difference was observed among the OE1, OE2, and WT (Additional File 3). Whereas on 1/2 MS plates containing caffeine, OE1 and OE2 grew better than WT, especially for the concentration of 0.03% and 0.05%. Transgenic plants showed longer taproots and more lateral roots. In addition, the toxic effect on Arabidopsis was observed in the presence of caffeine. All of the Arabidopsis lines were obviously inhibited under caffeine treatments, and the rosette leaves even showed visible symptoms of chlorosis in the plates containing 0.03% and 0.05% caffeine (**Figure 9A**).

To investigate the amounts of caffeine accumulated in the aerial parts of Arabidopsis, we cultivated plants on 1/2 MS medium with 0.02% caffeine for 21 days. Compared with WT, CsPUP10.1 transformants (OE1 and OE2) accumulated more caffeine (**Figure 9B**). The caffeine transport capacities in transgenic lines were improved for 79% and 45% in OE1 and OE2, respectively. These findings were consistent with the heterologous expression system in yeast and strongly demonstrated the function of CsPUP10.1 in caffeine transportation.

## 4 Discussion

PUPs are specifically found in vascular plants. Previous studies of PUPs in plants were mainly focused on their transport functions in adenine and cytokinin (Jelesko 2012) Because of their structural similarities to caffeine, a yeast mutant *fcy2* in caffeine transport deficiency was generally used as model cells for complementary assay. Generally, there is competitive inhibition between these purine-ring substrates. Therefore, more PUP members were proved to be cytokinin transporters in plants (Kang et al., 2017). AtPUP1 and AtPUP2 showed transport activity for adenine, cytokinin, and caffeine in *fcy2* yeast (Gillissen et al., 2000; Bürkle et al., 2003). OsPUP7 was proved to transport caffeine in *fcy2* yeast, which provides convincing evidence for its important role in cytokinin transport (Qi and Xiong, 2013). CcPUP1 and CcPUP5 were demonstrated to facilitate the transport of adenine, but not caffeine (Kakegawa et al., 2019). Recently, an expanding role of PUPs in the transportation of both purine-alkaloids and non-purine-alkaloids was also identified (Jelesko, 2012).

Here, eight CsPUP members were identified in tea plants and named by their homology with Arabidopsis (**Table 1**). The amino acid sequences of CsPUPs were highly conserved. Ten TMDs were detected in most members, which may have an important effect on protein function (**Figure 1**). Consistent results were also found in Arabidopsis, maize, and rice, implying similar transport function of CsPUPs in tea plants (Qi and Xiong, 2013). The CsPUP members could be divided into classes I, II, and III by phylogenetic analysis. PUPs with higher sequence similarity in different species were clustered into one group. Whereas, gene members from the same species showed further genetic distance on the evolutionary tree (**Figure 2**). The eight CsPUPs were randomly distributed on six chromosomes in the tea genome (**Figure 3**). These results indicated that both sub-functionalization and neo-functionalization occurred during the evolution of gene and species (Jelesko, 2012). The function of CsPUPs transporters in tea plants were also expected to diversified considerably. Analysis of gene expression in different tissues revealed that CsPUPs were mainly expressed in the aerial parts of tea plants. The expression levels of CsPUP1, CsPUP3.1, CsPUP3.3, CsPUP10.1, and CsPUP10.2 were higher in leaves, whereas CsPUP3.2 and CsPUP4 showed the highest expression level in stems (**Figure 4**). PUPs in other plants were also reported to have different expression characteristics. AtPUP1 was highly expressed in leaves, stems, and flowers (Gillissen et al., 2000). AtPUP2 was mainly expressed in the vascular tissue of leaves. The expression of AtPUP3 was restricted in pollen (Bürkle et al., 2003). AtPUP14 was widely expressed in all tissues and organs (Zürcher et al., 2016). The differences of gene expression patterns implied variations of protein function in another way. Previous studies revealed that gene expression patterns were closely correlated with the distribution of their substrates in plants. NtNUP1 showed the greatest expression level in root tips, which is the main organ nicotine biosynthesis. Further study proved that NtNUP1 mediated the uptake of nicotine in root cells and thus affected its accumulation in tobacco (Hildreth et al., 2011). There are abundant codeine and morphine in the latex of opium poppy. PsBUB1 was also highly expressed in the latex and functioned as benzylisoquinoline alkaloid transporters (Dastmalchi et al., 2019). Distinct from the alkaloid distributions in tobacco and opium poppy, caffeine in tea plants was found primarily in leaves, followed by stems and roots (Facchini, 2001). Therefore, CsPUP1, CsPUP3.1, and CsPUP10.1, which showed higher expression level in leaves, were selected for further investigation. The results of correlation analysis revealed that CsPUP1, CsPUP3.1, and CsPUP10.1 were significantly negatively correlated with caffeine content (**Table 2**). It suggested their potential roles in caffeine metabolism.

The complementation assay in the yeast and subcellular localization analysis demonstrated that CsPUP1, CsPUP3.1, and CsPUP10.1 functioned as caffeine influx transporters in plasma membrane (**Figures 7**, **8B**). This functional mode of CsPUPs was coincident with their homologous genes in other plants. AtPUP1 was localized on plasma membrane. It was the first member to be reported to promote the caffeine uptake in the yeast expression system (Gillissen et al., 2000; Szydlowski et al., 2013). NtNUP1 was primarily localized on plasma membrane and showed nicotine-specific uptake activity (Hildreth et al., 2011). In opium poppy, six of the nine PsBUPs facilitated the transportation of alkaloids and increased their yields when expressed in host yeast. PsBUP1 was found to be a laticifer-specific plasma membrane transporter (Dastmalchi et al., 2019). OsPUP7 was proved to transport caffeine into cells through complementary experiment in the yeast *fcy2* mutant (Qi and Xiong, 2013).

For most of the species without any alkaloids produced, exogenous alkaloid has inhibitory and toxic effects on their growth and development. Whereas for the plants producing such metabolites, they develop characteristic self-tolerance mechanisms and usually are insensitive to alkaloids (Shitan, 2016). This phenomenon was demonstrated in Arabidopsis, tobacco, pea, and yeast (Bard et al., 1980; Mohanpuria and Yadav, 2009b; Curlango-Rivera et al., 2010; Alkhatib et al., 2016). Consistent phenotype was also observed in our study. Interestingly, compared with the blank group, repressed growth of transformants could be partly rescued by CsPUPs (**Figures 8A**, **9A**). This phenomenon was distinct from studies on non-caffeine plants. Transgenic yeasts expressed AtPUP1 and OsPUP7 showed worse growth conditions than control under exogeneous caffeine treatment (Bürkle et al., 2003; Qi and Xiong, 2013). Transgenic *fcy2* yeast with CcPUP1 and CcPUP5 also showed more pronounced inhibited phenotype than control (Kakegawa et al., 2019). Whereas, yeast transformants with CsPUP1, CsPUP3.1, and CsPUP10.1 exhibited better growth conditions than control in media containing caffeine (**Figure 8A**). Similar phenotype was also confirmed in CsPUP10.1-over-expressed Arabidopsis lines (**Figure 9A**). It suggested that CsPUPs functioned distinctively in caffeine transport in tea plants.

Recently, functional identification of theanine transporters in tea plants was reported. The complementation assays of amino acid permeases showed that they could transport theanine into yeast cells as nitrogen source. Thus, transgenic yeast exhibited alleviated toxicity and better growth condition than control under theanine treatment (Dong et al., 2020). Moreover, transformant yeast with AtPUP1 imported exogenous adenine into cells as nitrogen source and grew better than control. Further studies revealed that AtPUP1 functioned as a cytokinin transporter and was involved in its retrieval from the xylem sap (Bürkle et al., 2003). Moderate exogenous caffeine was proved to positively regulate plant growth in sunflower and tobacco (Khursheed et al., 2009; Alkhatib et al., 2016). In addition, the absorption and degradation of exogenous caffeine were also revealed in wheat, barley, poplar, and lettuce (Pierattini et al., 2016; Yahyazadeh et al., 2017; Chuang et al., 2019; Broughton et al., 2020; Vannucchi et al., 2020). Caffeine in tea leaves could be slowly catabolized along with the growth and development of plants (Mohanpuria et al., 2009a; Ashihara et al., 1997; Zhu et al., 2019). Some microorganisms were also reported to degrade caffeine and its derivates as nitrogen source (Mazzafera, 2002; Mazzafera, 2004; Dash and Gummadi, 2006). These studies indicated that part of exogenous caffeine could be decomposed and recycled as nitrogen source in different organisms to maintain their survival and growth.

Furthermore, in addition to that in the plasma membrane, abundant and stronger GFP signals of CsPUPs were also observed in the form of hollow or solid dots and distributed widely in cells (**Figure 7**). According to the shape and allocation, it was speculated that they were whether vesicles, peroxisomes, or lysosomes (Uemura et al., 2004; Winters et al., 2020). The characteristic localization patterns of these CsPUPs implied that they might shuttle between the organelles and function distinctly in tea plants from other species.

Intracellular and extracellular vesicles play an essential role in the transportation and compartmentation of secondary metabolites (Shoji et al., 2014; Zhao et al., 2020). The transport vesicles could bound to membrane and carry substrates from the organelle (generally ER) where their biosynthesis takes place to another organelle (generally vacuoles) (Bonifacino and Glick, 2004). Vacuoles can be divided into two types: lytic vacuoles (LVs) and protein storage vacuoles (PSVs). Hydrolases in LVs degrade unwanted cellular substances. Whereas, PSVs accumulate abundant substances for storage (Tan et al., 2019). The final distribution patterns of metabolites vary with cell types, tissues, developing stages, and plant species (Shitan and Yazaki, 2007; Shitan and Yazaki, 2020; Gani et al., 2021). This transport mechanism was widely demonstrated in flavonoid accumulation in plants (Zhao and Dixon, 2009; Zhao, 2015; Pucker and Selmar, 2022). Hyper-accumulated anthocyanin in purple tea leaves was also proved to be closely related with active vesicular trafficking (Wei et al., 2019).

Peroxisome is a circular organelle and widely distributes in nearly all eukaryotes. The shape of peroxisome could be changed from sphere to ellipse along with plant growth and environmental conditions (Beevers, 1979). This process is closely associated with diverse metabolic pathways in plants, including catabolism of polyamines, biosynthetic pathway of hormones, and alleviation of the toxicity of reactive oxygen species and other harmful products (Oikawa et al., 2019).

Lysosome is another round organelle and displays as vesicular structure in eukaryotes. The lysosome system is one of the powerful hydrolytic mechanisms involved in the degradation of cellular components (Lloyd, 1996). Numerous metabolite transporters were discovered in the lysosome membrane. The degradation products of nutriments, such as amino acids, could be transported by these transmembrane proteins for recycle and reutilization (Tong et al., 2010).

To date, the exact subcellular organelle of caffeine synthesized, stored, and degraded remains unclear. The site of caffeine biosynthesis seemed to vary with species. Caffeine biosynthesis in tea plants was closely associated with chloroplasts (Ashihara et al., 2013). Whereas, caffeine in coffee appears to be synthesized in the cytoplasm (Ogawa et al., 2001; Kumar et al., 2007). Vacuole was a putative location for caffeine storage in plants (Wink, 2010).

**Figure 10:**
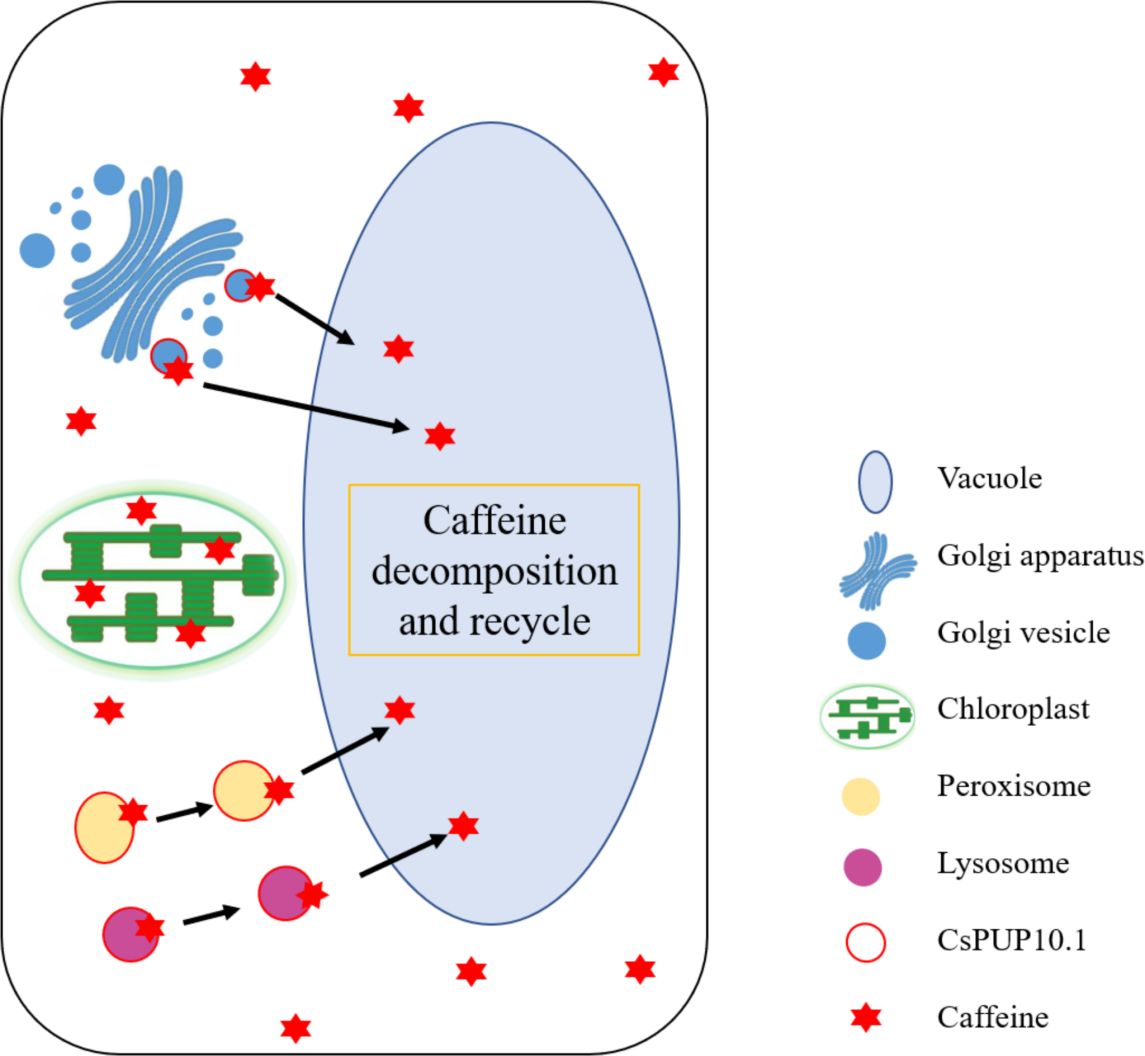
Putative mechanism of CsPUP10.1 in caffeine transport.

Therefore, it can be inferred that CsPUPs functioned as caffeine transporters in two ways in heterologous expression systems. For one thing, CsPUPs on plasma membrane imported the external caffeine into cells, which caused more caffeine accumulation in the transgenic yeast and Arabidopsis than control. For another thing, deposited caffeine in cells could be comparted and transported by CsPUPs. Then, part of caffeine could be degraded and converted into a form of nitrogen source for further utilization in vacuole (**Figure 10**). Thus, transformants with CsPUPs showed better growth conditions and enhanced tolerance to caffeine. For tea plants with endogenous caffeine produced, caffeine was widely distributed in leaf cells. CsPUPs showed extended subcellular localization and distinct phenotypic differences in heterologous expression systems. These results implied characteristic mechanism of caffeine transport in tea plants. Compartmentalized caffeine in tea plant cells could be delivered by CsPUPs from the site of synthesis or storage to the other organelles for decomposition and further utilization. In conclusion, these differences better explain the distinctive mechanisms of CsPUPs in caffeine transport in tea plants.

In summary, in total, eight PUP members from the whole genome of tea plant were identified. Then, bioinformatics and expression patterns of these genes were analyzed to determine their potential roles in caffeine metabolism. Among of them, CsPUP1, CsPUP3.1, and CsPUP10.1 showed higher expression level in ML and were significantly negatively correlated with caffeine content. Complementation assays in yeast revealed that CsPUP1, CsPUP3.1, and CsPUP10.1 functioned as caffeine transporters. Further heterologous expression analysis in Arabidopsis re-confirmed the function of CsPUP10.1. Combined with the results of subcellular localization and expression mode, it suggested that CsPUPs play an important role in the intracellular transport of caffeine in tea plants. Deposited caffeine in tea leaves might be transported by CsPUPs among different subcellular organelles for facilitating its metabolism. This study provides a new insight into the molecular mechanisms of caffeine regulatory network in tea plants.

## Data availability statement

The original contributions presented in the study are included in the article/**Supplementary Material**. Further inquiries can be directed to the corresponding author.

## Author contributions

Conceived and designed the experiment: KW, HC, and LW. Performed the experiments: YZ, LG, and YL. Analyzed the data: YZ, KW, LG, and LW. Contributed reagents, materials, and analysis tools: HC, LW, and CC. Contributed to the writing of the manuscript: YZ, KW, and LW. All authors contributed to the article and approved the submitted version.

## Supplementary material

Additional file 2 The overall expression levels of CsPUPs in different tissues.

Additional file 3 Growth conditions of Arabidopsis on normal 1/2 MS medium.
