# Supplementary figures and images for: Functional identification of purine permeases reveals their roles in caffeine transport in tea plants (Camellia sinensis)
Source: Front Plant Sci. 2022 Dec 15;13:1033316. doi: 10.3389/fpls.2022.1033316 (PMC9798130; doi:10.3389/fpls.2022.1033316)

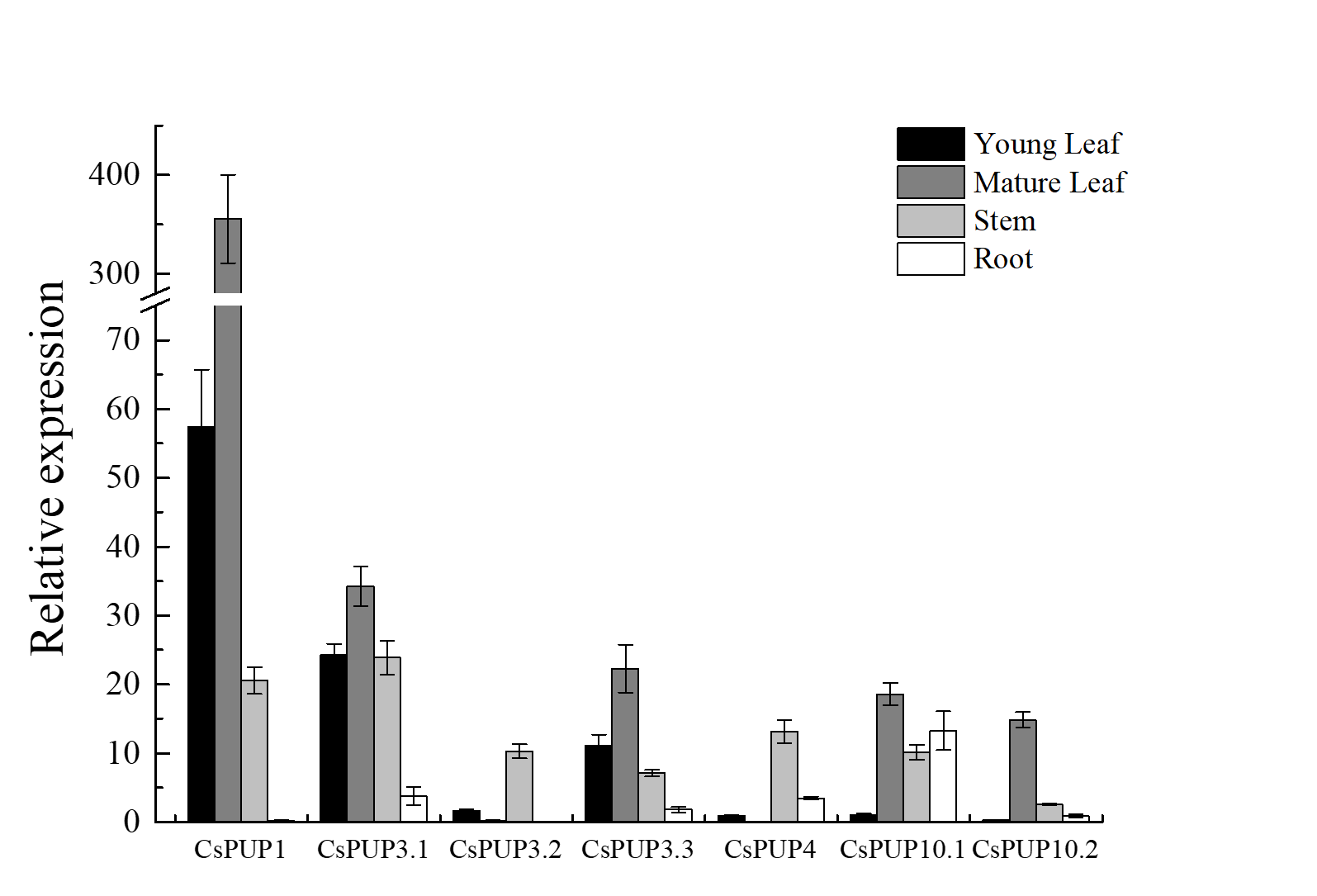

Supplement: Additional file 2 — The overall expression levels of CsPUPs in different tissues. [file Image_1.tif]

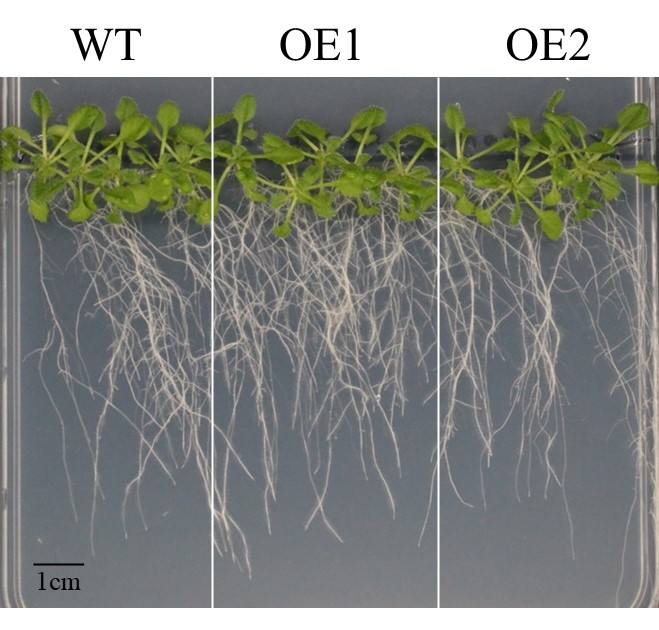

Supplement: Additional file 3 — Growth conditions of Arabidopsis on normal 1/2 MS medium. [file Image_2.tif]
